# A case report of combined methylmalonic acidemia and homocysteinemia presented with cerebral sinus thrombosis and fluctuating cognitive impairment

**DOI:** 10.1097/MD.0000000000042919

**Published:** 2025-06-13

**Authors:** Jingru Wang, Lu Liu, Huiting Wang

**Affiliations:** a Department of Neurology, Liaocheng People’s Hospital, Liaocheng, Shandong Province, PR China; b Department of Neurology, Liaocheng People’s Hospital, Shandong University, Jinan, Shandong, PR China.

**Keywords:** C, c.609G>A, cognitive impairment, erebral sinus thrombosis, methylmalonic acidemia, MMACHC

## Abstract

**Rationale:**

MMACHC deficiency leads to combined methylmalonic acidemia and homocysteinemia. The disease is characterized by the presence of methylmalonic acidemia and hyperhomocysteinemia, leading to widespread clinical manifestations affecting multiple organs. Due to the low incidence of cblC deficiency and the diversity of its clinical phenotypes, diagnosis was challenging and often results in delays or missed diagnoses. We now report a case of a male patient who experienced fluctuating cognitive impairment and headaches for 3 times. He was initially diagnosed with encephalitis and venous sinus thrombosis, but his symptoms recurred. Later, significantly elevated levels of homocysteine and methylmalonic acid were detected. Genetic analysis confirmed the presence of a heterozygous mutation in the MMACHC gene, establishing the definitive diagnosis. This case is reported due to its rarity and diverse clinical presentations, highlighting the need for increased awareness of this condition.

**Patient concerns:**

A male patient exhibited recurrent episodes of fluctuating cognitive impairment and headaches.

**Diagnoses:**

The patient underwent magnetic resonance imaging, lumbar puncture, and cerebrospinal fluid analysis. To confirm the diagnosis, genetic sequencing analysis was also conducted.

**Interventions:**

In terms of intervention, the patient received supplementation with cobamamide, vitamin B1, and folic acid, leading to gradual relief and improvement of symptoms.

**Outcomes:**

The patient’s headaches and cognitive impairment symptoms have alleviated, and there have been no recurrences during follow-up.

**Lessons:**

To our knowledge, there are few clinical cases of methylmalonic acidemia presenting primarily with episodic cognitive impairment and venous sinus thrombosis. Therefore, it is essential to enhance the recognition and differential diagnosis of such symptoms to improve the accuracy and speed of the disease diagnosis.

## 1. Introduction

Methylmalonic acidemia (MMA), also referred to as methylmalonic aciduria, is an autosomal recessive disorder of organic acid metabolism. It primarily results from defects in the enzyme methylmalonyl-CoA mutase or deficiencies in its cofactor, adenosylcobalamin (vitamin B12). This enzymatic dysfunction leads to the abnormal accumulation of metabolites, including methylmalonic acid, propionic acid, and citric acid esters in the bloodstream, which can result in severe clinical manifestations such as pancreatitis, renal failure, and neurological symptoms like mental disturbances, optic atrophy, and signs associated with spinal cord and basal ganglia damage.^[1]^ MMA is linked to mutations in the MMACHC gene, located at chromosome 1p34.1, which impact the function of the enzyme and lead to reduced intracellular production of vitamin B12, essential cofactors for methylmalonyl-CoA mutase and methionine synthase. The deficiency of these enzymes contributes to elevated levels of methylmalonic acid and homocysteine within the body. Clinically, MMA is categorized into early-onset and late-onset forms. Early-onset cases typically manifest within the first year of life, while late-onset cases may present symptoms between ages 4 to 14 and can even extend into adulthood, often involving multi-system complications. Late-onset MMA is less frequently reported, and its wide-ranging symptoms can lead to significant clinical heterogeneity. Misdiagnosis is common, especially in the absence of a family history. We report a case of late-onset methylmalonic acidemia presenting with recurrent cognitive fluctuations and cerebral venous thrombosis as the primary symptoms, which differ from those typically described in the literature. This case highlights the unique clinical manifestations of the condition and aims to contribute to the understanding and early diagnosis of similar cases in the future.

## 2. Case description

A 13-year-old male patient, presented to our hospital on December 26, 2016, with a chief complaint of cognitive decline that had persisted for over a month, worsened by headaches that had intensified over the past 10 days. His family reported noticing a marked decrease in his responsiveness and learning ability, as well as diminished verbal communication and social interaction. The patient had been unable to complete his homework. Approximately 10 days prior to his admission, he had fallen while playing with classmates, landing on his head; although there was no apparent external injury at the time, he subsequently experienced persistent headaches. His medical history was unremarkable, and he was born via uncomplicated term delivery, demonstrating normal speech and physical activity in line with his peers. Until this incident, his academic performance had been satisfactory, generally ranking above average.

**Figure 1.**
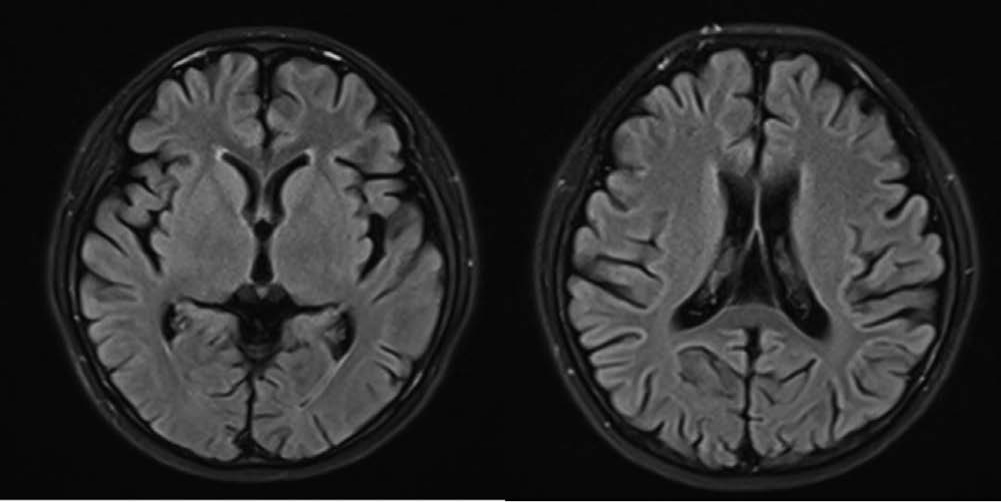
Brain MRI (2016): Mild brain atrophy. MRI = magnetic resonance imaging.

After admission, the physical examination revealed that the patient was alert but exhibited difficulties with fluency in speech, along with impaired orientation, memory, and calculation abilities. Other physical exam findings were within normal limits. Subsequent investigations included routine blood tests and comprehensive biochemical analyses, which indicated elevated homocysteine levels at 48.1 µmol/L, while other parameters remained normal. Magnetic resonance imaging (MRI) showed mild cerebral atrophy (Fig. 1). Based on these findings, a diagnosis of viral encephalitis was made. The patient was treated with a regimen that included vitamin B12, vitamin B6, and vinpocetine injection, aimed at providing anti-inflammatory and symptomatic relief. Following treatment, the patient demonstrated significant clinical improvement, regaining normal communication abilities, as well as enhanced calculation, orientation, and comprehension skills before being discharged.

**Figure 2.**
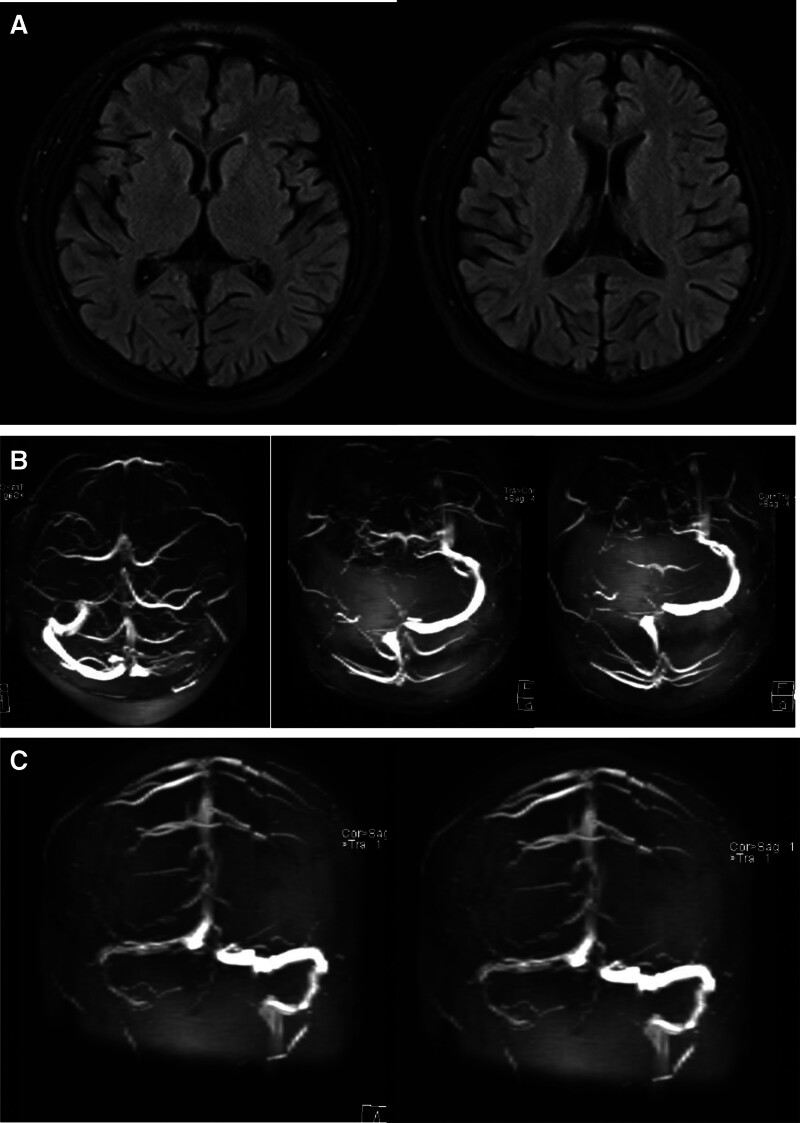
(A) Brain MRI (2021): mild brain atrophy, showing slight reduction in brain atrophy compared to the previous scan in 2016. (B) (2021) MRV: there was a significant likelihood of venous thrombosis in the right transverse sinus, sigmoid sinus, and the lumen of the internal jugular vein. The enhancement of the superior sagittal sinus lumen was locally diminished, which does not exclude the possibility of local thrombosis or slow venous. (C) Follow-up MRV after treatment: the right transverse sinus and superior sagittal sinus lumen show improvement compared to previous scans, while the right sigmoid sinus and internal jugular vein appear largely unchanged. MRI = magnetic resonance imaging, MRV = magnetic resonance venous imaging.

After discharge, the patient initially maintained normal motor function and performed well academically. However, during his time in high school, his academic performance began to decline, ultimately leading him to enroll in a vocational technical school. On December 3, 2021, at the age of 18, he was readmitted to the hospital with complaints of decreased memory, emotional disturbances, sluggishness for over ten days, and headaches persisting for the last 3 days. His teachers and peers at the vocational school observed significant memory issues and unusual emotional behavior, characterized by impaired learning ability during classes, difficulties recalling recent events, irritability, reduced social interaction, and decreased responsiveness, including instances of not answering questions. Notably, for the 3 days prior to admission, the patient reported headaches, which he could not precisely describe, and he did not present with fever. Upon admission, his physical examination revealed that he was alert but displayed reduced mental vitality, limited facial expressions, and impaired memory and calculation abilities. He was partially uncooperative during the examination, which hindered thorough evaluation of motor strength and muscle tone. The examination showed equal and round pupils measuring 3 mm, with a positive light reflex; ocular movements were intact without nystagmus, and facial features (including nasolabial folds) were symmetric. Coordination, deep and superficial sensory tests could not be performed, and signs of meningeal irritation were negative. MRI showed abnormal signal in the right transverse sinus. The findings also indicated inflammation of the right mastoid process, while comparisons with the MRI from December 2016 revealed slight enlargement of the ventricular spaces but notable improvement in the width of the brain sulci (Fig. 2A). Further magnetic resonance venous imaging (MRV) investigation suggested significant thrombosis in the right transverse sinus, sigmoid sinus, and internal jugular vein, with a possibility of local thrombus formation or slowed venous flow in the superior sagittal sinus (Fig. 2B). A computed tomography (CT) scan of the mastoid revealed findings consistent with right mastoiditis, while a chest CT indicated inflammatory changes in the left lower lobe. Cerebrospinal fluid (CSF) analysis revealed normal cell counts with negative protein levels, and the CSF was clear with an intracranial pressure of 250 mm H_2_O. Blood tests showed mild anemia with a red blood cell count of 3.98 × 10^12^/L and hemoglobin of 117.00 g/L, along with increased homocysteine levels at 100.00 µmol/L. The erythrocyte sedimentation rate was elevated at 48 mm/h. The patient was unable to complete the Mini-Mental State Examination (MMSE) and Montreal Cognitive Assessment due to cognitive impairments. The primary diagnoses included cerebral venous sinus thrombosis, mastoiditis, and bacterial pneumonia. Treatment commenced with low molecular weight heparin for anticoagulation, as well as antibiotics for the infection, alongside measures to improve circulation and promote brain metabolism. Supportive care included the administration of folic acid, methylcobalamin, and vitamin B6. Follow-up imaging, including a repeat CT of the mastoid, confirmed the presence of mastoiditis. After a course of treatment, subsequent MRV revealed improvement in the patency of the right transverse and superior sagittal sinuses compared to the earlier studies, with the right sigmoid sinus and internal jugular vein remaining relatively unchanged (Fig. 2C). Upon discharge, the patient exhibited significant symptomatic improvement; his memory was markedly better, and he responded fluently during interactions, with no reports of headaches or fever. Neurological examination at discharge showed he was alert and responsive, with improved memory and calculation abilities. The MMSE score at discharge was 25. Post-discharge, he was prescribed rivaroxaban and cefixime for ongoing management.

**Figure 3.**
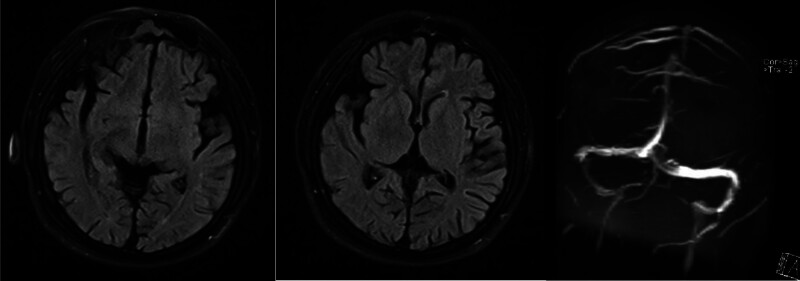
2022 MRI + MRV: mild cerebral atrophy; the blood flow signals in the right sigmoid sinus and internal jugular vein lumen were slightly weaker, with a possible localized narrowing at the terminus of the sigmoid sinus. The enhancement of the superior sagittal sinus lumen was locally diminished, showing significant improvement. MRI = magnetic resonance imaging, MRV = magnetic resonance venous imaging.

**Figure 4.**
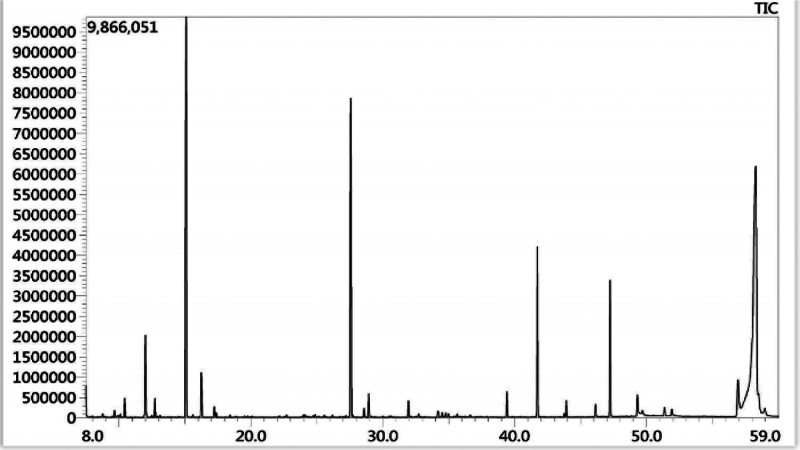
Elevated methylmalonic acid (methylmalonic acid-2 level: 96.6), indicating the presence of methylmalonic acidemia.

**Figure 5.**
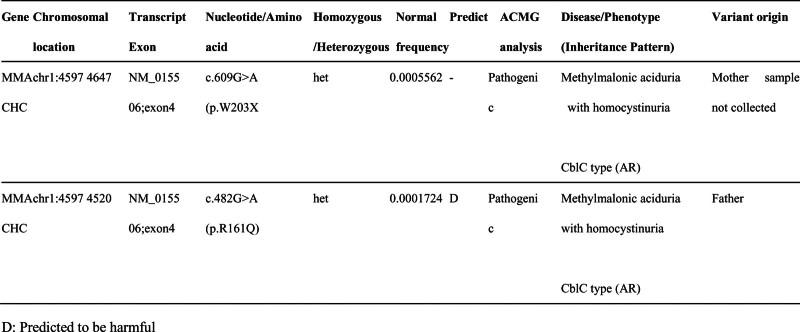
Identification of 2 heterozygous mutations in the MMACHC gene. The mutation c.609G > A (p.W203X) is not present in the patient’s father at this locus, while the mutation c.482G > A (p.R161Q) is a heterozygous variant in the patient’s father.

**Figure 6.**
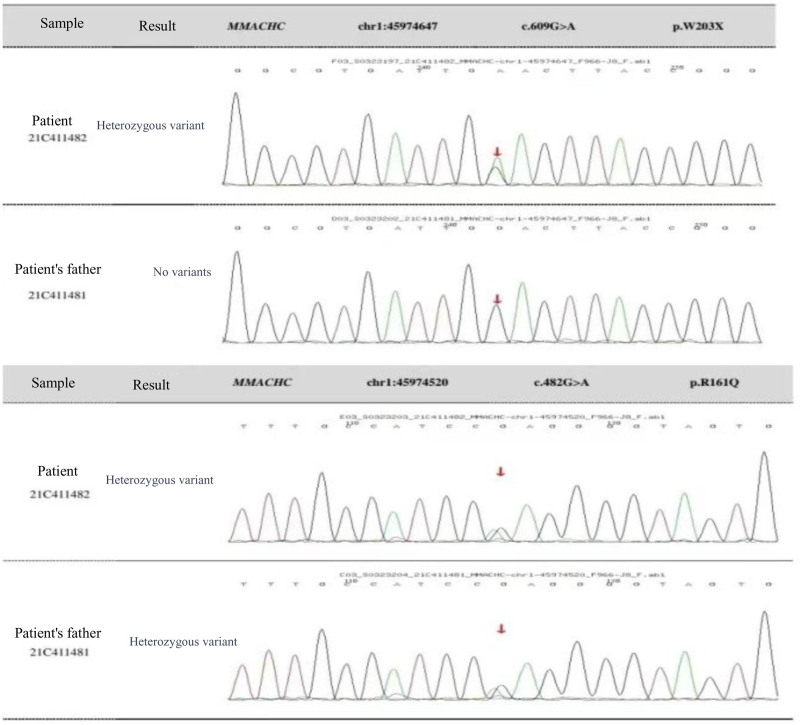
Sequencing chromatograms of the patient and their father for the mutations c.609G > A and c.482G > A.

**Figure 7.**
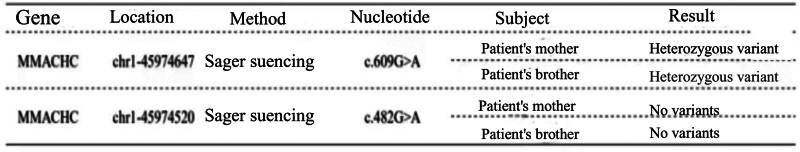
The mutation c.609G > A (p.W203X) is a heterozygous variant in both the patient’s mother and brother, while the mutation c.482G > A (p.R161Q) is not present in either the patient’s mother or brother.

**Figure 8.**
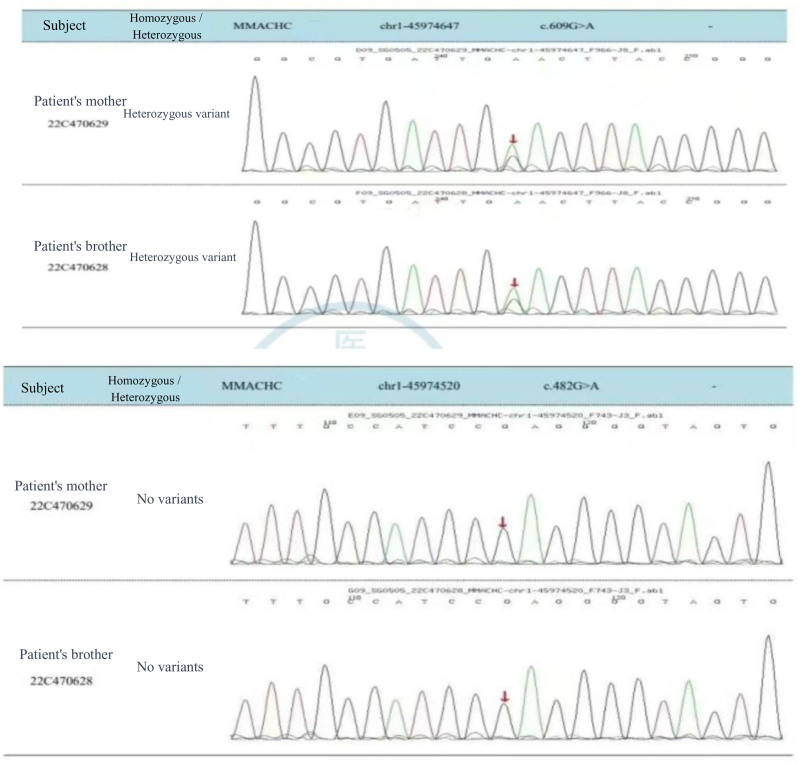
Sequencing chromatograms for the patient’s mother and brother displaying the mutations c.609G > A and c.482G > A.

On January 26, 2022, the patient was admitted to the hospital for the third time, having experienced a recurrence of emotional disturbances and sluggishness for the prior 4 days. He reported weakness in his lower extremities, which led to difficulty walking and tremors in both legs while standing. Additionally, he exhibited signs of apathy, low mood, and poor appetite. Importantly, he did not experience dizziness, headaches, slurred speech, gait disturbances, or loss of consciousness. Upon examination, the patient was alert but showed decreased mental activity and limited facial expression. He struggled with computation and exhibited significant memory deficits, particularly with recent information, while orientation to place was relatively intact; however, his orientation to time was poor. Neurological assessment indicated normal muscle strength in the upper and lower limbs, but increased muscle tone in the lower extremities, with deep tendon reflexes rated at +++. Some pathological reflexes were noted on both sides (±). Coordination tests in the lower limbs showed deviations, although there were no significant abnormalities in sensory examinations. MMSE was not able to be completed due to the patient’s condition. Repeat imaging using MRI and MRV revealed mild cerebral atrophy and noted a slight decrease in blood flow signals in the right sigmoid sinus and internal jugular vein, with mild stenosis at the termination of the sigmoid sinus. Comparison with previous imaging from December 2021 indicated improvements (Fig. 3). Further analysis of homocysteine levels revealed a reading of 99.90 µmol/L. A lumbar puncture was performed, yielding clear CSF with an intracranial pressure of 180 mm H_2_O, and biochemical analysis showed no abnormalities. Following admission, the patient was treated with measures to improve circulation and nutritional support. However, his symptoms gradually worsened, leading to an inability to walk independently and demonstrating a scissors gait. Consequently, he was referred for further evaluation at Beijing 301 Hospital, where metabolic testing, including blood and urine organic acid assessments, was conducted (Fig. 4). Genetic testing confirmed elevated levels of methylmalonic acid, indicating a diagnosis of MMA. Further genetic analysis revealed 2 heterozygous mutations in the MMACHC gene (c.609G > A and c.482G > A), confirming a cblC type of MMA (Figs. 5 and 6). Upon recognizing the need for familial assessment, the patient’s mother and brother were also encouraged to undergo genetic testing (Figs. 7 and 8). Once a definitive diagnosis was established, the patient was treated with methylcobalamin, vitamin B1, and folic acid, which led to an improvement in symptoms, allowing for discharge. Post-discharge, he was instructed to continue with intramuscular injections of methylcobalamin and oral vitamin B1 and folic acid. The patient gradually showed significant improvements in his condition, with normalized language and walking abilities. Follow-up tests indicated a decrease in homocysteine levels, which eventually returned to normal by the 6-month post-discharge follow-up. Notably, his MMSE score improved to 29, reflecting enhanced cognitive function and overall well-being.

## 3. Discussion

MMA, also referred to as methylmalonic aciduria, is characterized by elevated levels of methylmalonic acid in blood and/or urine, which may occur alone or in conjunction with other biochemical abnormalities, such as increased homocysteine and decreased methionine levels. MMA with hyperhomocysteinemia (combined MMA) is the predominant type observed in China^[2]^, accounting for approximately 80% of cases.^[3]^ The genetic basis for MMA involves mutations in the MMACHC gene. Many previous studies had reported mutations in the MMACHC gene, mostly as case reports. A new meta-analysis summarized the genetic variation information of 926 MMA patients in China; among them, 517 cases were combined MMA, and almost all combined MMA cases were caused by mutations in the MMACHC gene (cblC type). The c.609G > A variant was the most common in cblC type children, accounting for 43.01%, followed by the c.658_660delAAG, c.482G > A, c.80A > G, and c.394C > T variants.^[4]^ MMACHC gene encodes a protein believed to play a critical role in the intracellular transport of cobalamin and the biosynthesis of adenosylcobalamin and methylcobalamin. Mutations in the MMACHC gene impair the synthesis of these critical cofactors, leading to elevated levels of methylmalonic acid and homocysteine, reduced methionine production, and the subsequent accumulation of abnormal metabolites such as methylmalonic acid, hydroxybutyric acid, and citric meconate. This metabolic disruption can result in damage to multiple systems, including the nervous system, liver, kidneys, and hematological systems^[3,5,6]^.

The clinical manifestations of MMA exhibit considerable variability. Early-onset cases often present with acute deterioration, metabolic acidosis, and hyperammonemia shortly after birth, while later-onset cases may emerge at any point throughout life, presenting a diverse array of symptoms.^[2,7,8]^ Prior literature has documented primary neurological manifestations, such as cognitive impairment, behavioral disturbances, seizures, and signs of pyramidal tract dysfunction.^[9,10]^ Other reports have emphasized hematological and renal complications as dominant features.^[11,12]^ The variability of clinical presentation is compounded by the lack of specificity in symptoms, making it essential to rely on acylcarnitine profiles and urine organic acid analyses for accurate diagnosis, with genetic testing serving as the most reliable method for phenotypic classification.

A broad clinical heterogeneity among MMA patients may be attributed to the nature of various MMACHC mutations, alongside polymorphisms in other genes related to cobalamin metabolism.^[13]^ Environmental factors, ethnicity, dietary influences, and individual genetic backgrounds are also likely to contribute to the diversity of clinical outcomes. In this particular case, additional genetic testing for the MTHFR gene revealed a homozygous mutation at the 677 position. Previous studies suggest that MTHFR genotype appears not to significantly influence the age of onset, clinical phenotype, or prognosis in patients with cblC deficiency, although further investigation is warranted to clarify potential interactions.^[14]^ Interestingly, the phenomenon of recurrent cognitive impairment and venous sinus thrombosis has not been previously documented in the context of MMA.

The patient first presented at the age of 13. Although MRI indicated cerebral dysgenesis, due to their young age and the rapid alleviation of sluggishness, further examination and treatment were not pursued. During the second visit, the patient reported headache symptoms, and subsequent MRI revealed venous sinus thrombosis. The administration of anti-inflammatory, anticoagulant, and vitamin B complex therapies led to a rapid improvement in symptoms. Follow-up MRI showed significant improvement in the venous sinus thrombosis, at which point a definitive diagnosis was made, and no further analysis was conducted. Upon retrospective analysis, it was noted that both intravenous and oral forms of methylcobalamin and vitamin B complex were used during these 2 clinical treatments, which may be related to the improvement in clinical symptoms. However, the short interval before the third hospitalization and the severity of clinical symptoms may be related to the discontinuation of these medications and dietary status. Elevated homocysteine levels were consistently detected during all 3 hospitalizations, and while symptomatic treatment was provided, the relationship between the elevation of homocysteine and the clinical symptoms was not further investigated, complicating the diagnosis. Hyperhomocysteinemia is an independent risk factor for thromboembolism, which may directly contribute to the occurrence of venous thrombotic disease, such as venous sinus thrombosis in this case.^[3]^ The patient’s cognitive impairment is generally attributed to neurotoxicity caused by the accumulation of abnormal metabolic products in the blood. Increased homocysteine levels, impaired methyl metabolism, and oxidative stress play significant roles in the pathophysiological processes involved.^[6,15]^ Unlike previously reported cases, the cognitive impairment observed in this patient was characterized by its fluctuating nature and recurrent episodes. This pattern is thought to be related to the patient’s dietary structure. A detailed history revealed that in all 3 episodes, dietary factors, particularly reduced food intake, were contributing triggers. The lack of vitamin intake in the dietary structure resulted in the accumulation of abnormal metabolic products, leading to neurotoxic manifestations. However, these triggers were initially only considered simple causes for the elevation of homocysteine levels. Previous literature has indicated that changes in dietary structure can lead to alterations in clinical symptoms and laboratory findings in MMA.^[16]^

The treatment strategy for MMA aims to promptly improve clinical manifestations, normalize serum methionine levels, and reduce total homocysteine and MMA levels. Long-term follow-up studies have shown that the severity of clinical symptoms and prognosis are often associated with the timing of diagnosis and initiation of treatment. In some patients, a return to a normal dietary structure has been observed to correlate with symptomatic improvement.^[15]^ However, despite receiving treatment, some patients with cblC deficiency may still experience varying degrees of complications.^[17]^ It is advisable to initiate treatment and provide health education as early as possible, especially before the onset of symptoms. In the case presented, the patient exhibited significant improvement in clinical symptoms following treatment with vitamin B complex, indicating that this individual belongs to the effective B12-responsive category, which is associated with a relatively favorable prognosis.

Additionally, we conducted genetic testing on the patient’s father, mother, and younger brother (Figs. 7 and 8). The mutation c.482G > A was inherited from the father, while the other mutation c.609G > A was inherited from the mother. The younger brother also carries the c.609G > A mutation, which is considered the most common cblC mutation in the Chinese population.^[18]^ Aside from the patient, neither the parents nor the brother exhibited any similar symptoms. We provided health education to the patient’s family and recommended that family members undergo preconception counseling prior to pregnancy. This includes prenatal diagnosis and, when necessary, third-generation in vitro fertilization with genetic screening to prevent the birth of children with congenital organic metabolic disorders.

So far, research reports both domestically and internationally have primarily focused on pediatric cases, with relatively few instances of onset in adolescents or adults. Moreover, the clinical manifestations of adult-onset cases tend to display complexity and non-specificity, which increases the difficulty of diagnosis. Although the incidence of this disease is quite low, it is crucial to consider late-onset methylmalonic acidemia combined with hyperhomocysteinemia in differential diagnosis for patients exhibiting neurological symptoms that are inconsistent with common neurological disorders, especially in cases involving cognitive impairment, psychiatric symptoms, unexplained thrombosis, and issues related to the pyramidal tract and peripheral nerves.

## Author contributions

**Data curation:** Lu Liu.

**Writing – original draft:** Jingru Wang, Huiting Wang.

**Writing – review & editing:** Huiting Wang.
